# A Rare Case of Gastric Intramural Hematoma in Recurrent Leukemia

**DOI:** 10.7759/cureus.21385

**Published:** 2022-01-18

**Authors:** Andrew Alabd, Christopher Deitch

**Affiliations:** 1 Internal Medicine, Cooper University Hospital, Camden, USA; 2 Gastroenterology, Cooper University Hospital, Camden, USA

**Keywords:** leukemia, computed tomography, intramural, endoscopy, abdominal pain, stomach, hematoma

## Abstract

Gastric intramural hematoma (GIH) is a rare disorder that usually arises secondary to another condition or intervention, and less often occurs without an identifiable cause. Computed tomography (CT), as performed in this case, is the diagnostic modality of choice. The treatment comprises conservative measures, a minimally invasive approach, or surgical intervention. We present a case of recurrent acute lymphoblastic leukemia (ALL) as a new etiology for GIH that was managed conservatively to highlight the importance of including GIH in the differential for a patient with ALL, a drop in hemoglobin level, and vague gastrointestinal symptoms.

## Introduction

Gastric intramural hematoma (GIH) is a rare disorder, with only a few cases reported to date [1]. There are several well-known etiologies identified in the literature as causes of this disorder. However, acute lymphoblastic leukemia (ALL) has not been previously reported as a cause of GIH. The case presented in this study highlights the importance of including GIH on the differential for a patient with ALL with an acute drop in hemoglobin and vague abdominal complaints. Investigation should be conducted using proper imaging techniques [2,3]. The choice of management approach should be based on the characteristics of the hematoma and the overall stability of the patient’s condition.

## Case presentation

A 62-year-old man was presented with a prior known diagnosis of Philadelphia chromosome-positive B-Cell acute lymphoblastic leukemia (Ph+ B-ALL). He completed treatment with multiple cycles of HyperCVAD (a chemotherapy combination including cyclophosphamide, vincristine sulfate, doxorubicin hydrochloride, and dexamethasone) with remission three months prior to his hospital admission. The patient presented to our institution after being transferred for concerns related to ALL relapse.

The patient’s laboratory investigation was remarkable for a white blood cell count of 30 × 103/µL with 62% blast cells, hemoglobin 11 g/dl, platelets 52 × 103/µL, and normal liver function tests. His hospital course was complicated by right knee pain and swelling. The subsequent incision and drainage were found to be negative for the septic joint. He was being treated with antibiotics for epididymitis and ​had a bone marrow biopsy that confirmed the replacement of Ph+ B-ALL.

**Figure 1:**
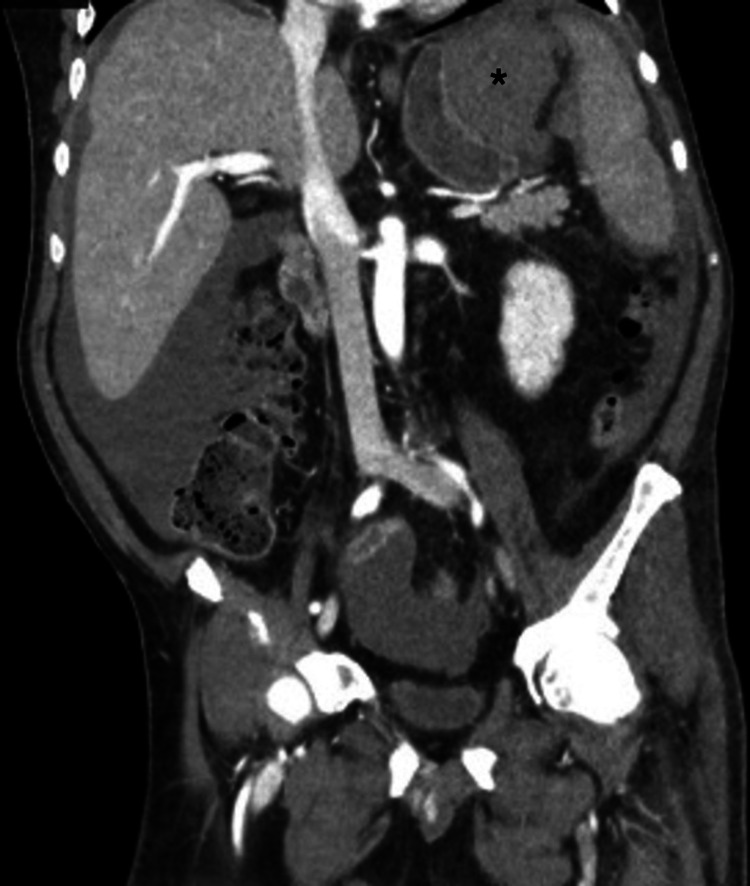
CT angiography image with gastric intramural hematoma marked with (*)

Over the following days, the patient began to complain of vague, generalized abdominal discomfort that was not associated with food intake. He had no nausea, vomiting, change in bowel habits, hematemesis, hematochezia, or melena. His routine daily laboratory examination was remarkable for an acute drop in his hemoglobin to 6.3 g/dl. Intravenous pantoprazole was initiated for a presumed upper gastrointestinal bleed of unknown origin. One unit of packed red blood cells raised the patient’s hemoglobin to appropriate levels. A CT angiography showed a large high attenuation structure adjacent to the greater curvature of the patient’s stomach measuring 11.7 cm × 6.7 cm × 6.0 cm, consistent with hematoma and with no contrast extravasation (Figure 1).

**Figure 2:**
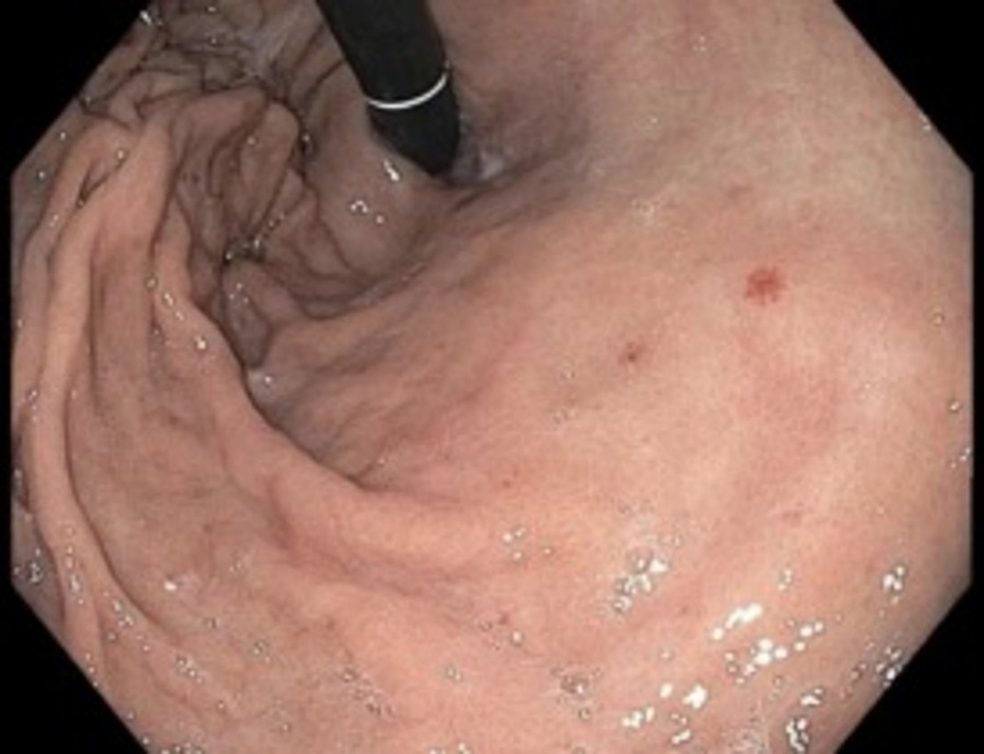
Endoscopic view of the gastric fundus

Once the patient was hemodynamically stable, an upper endoscopy was performed to evaluate for possible impingement on the pylorus or the presence of gastric lesions. The results showed non-bleeding angioectasias in the stomach. The remainder of the stomach was endoscopically normal (Figure 2). The patient had a blood transfusion with an appropriate response.

The patient was started on chemotherapy re-induction with the goal of remission and transplant evaluation. However, this approach was complicated by neutropenic fever and shock, which required vasopressors and a brief escalation of his care to the intensive care unit. He was treated for Clostridium difficile colitis, bacteremia, and liver abscess. A repeat computed tomography (CT) scan two weeks later showed a decrease in the size of the hematoma along the greater curvature of the stomach at 5.6 cm × 4.4 cm. He was able to complete chemotherapy induction and was discharged with plans for outpatient follow-up and transplant evaluation.

## Discussion

Intramural hematoma of the gastrointestinal tract is relatively rare, with hematomas having been reported throughout the gastrointestinal tract [1]. GIH is rare, with only a few cases reported to date [2,4]. In the stomach wall, the arteries and veins are arranged side by side and distributed in a mesh-like pattern with a significant number of blood vessels in the submucosal layer [5]. The shredding of terminal arteries at the point of leaving the mesentery and penetrating the muscular layer with subsequent dissection of the muscularis propria from the submucosa leads to hematoma formation in the submucosal or muscular layer [1,6].

GIH usually arises secondary to another condition or intervention. Spontaneous intramural hematoma of the stomach without an identifiable cause has been reported in the literature as well [7]. One of the main etiologies of gastric intramural hematomas is that of procedure-related cases, including argon plasma coagulation (APC), endoscopic mucosal resection (EMR), EUS-guided fine needle aspiration (EUS-FNA), extracorporeal shock wave lithotripsy (ESWL), percutaneous endoscopic gastrostomy (PEG), and post-injection therapy [6,8-13]. Other causes include the use of anticoagulants [14], ulcer disease [11], amyloidosis [15], and pancreatitis [16], or even in the setting of infection [17]. In addition, hematomas of various mucosal structures are well-known complications in patients with hemophilia. We report ALL as a new etiology for GIH that has not been reported previously in the literature to highlight the importance of assessing for GIH in a patient with vague abdominal pain and an unexplained drop in hemoglobin.

Barium studies are no longer used as a diagnostic modality for suspected GIH, given their inability to distinguish a hematoma from a solid tumor mass [4]. Evaluation of gastrointestinal intramural hematomas using ultrasound shows a tubular anechoic or hypoechoic mass containing a core of strong echoes. The nonspecific pattern of GIH that often mimics neoplasms or inflammatory lesions gives ultrasound a poor discriminatory capacity for gastric hematomas [4,18]. The CT scan is the current diagnostic modality of choice for gastrointestinal-wall hematomas given its ability to differentiate solids from liquids. Unlike neoplasms, gastrointestinal hematomas lack signs of calcification on CT imaging [3]. CT angiography can further illustrate active bleeding or extravasation, which makes it the appropriate modality to guide therapeutic intervention if needed [2]. Our patient’s CT imaging findings showed the hematoma formation with no extravasation and a decrease in hematoma size upon repeat imaging with stable hemoglobin levels.

The management of gastric hematomas largely depends on the presence of active bleeding, the patient’s hemodynamic stability, and, often, the etiology of the hematoma formation. GIH secondary to intrinsic coagulopathy is generally managed conservatively through blood and coagulation factor replacement [4]. Active extravasation of the contrast upon imaging often requires transcatheter arterial embolization (TAE) to control bleeding hematoma [2]. Other therapeutic options include percutaneous external drainage of the gastrointestinal hematoma [19], the use of lumen-apposing metal stents [20], and surgical intervention [15].

## Conclusions

Through a case study of a patient with a diagnosis of ALL, we highlight this condition as a new, previously unreported etiology for GIH, a rare disorder for which CT of the abdomen is the preferred diagnostic modality. A GIH formation should be suspected in a patient with ALL who has an acute drop in hemoglobin and vague abdominal complaints. GIH can usually be managed with a conservative approach and blood transfusion, while minimally invasive therapeutic interventions and surgery should be reserved for cases that do not respond to conservative approaches.
